# Evaluating relationships between seed morphological traits and seed dormancy in *Chenopodium quinoa* Willd.

**DOI:** 10.3389/fpls.2023.1161165

**Published:** 2023-10-20

**Authors:** Emma M. McGinty, Evan B. Craine, Nathan D. Miller, Cristina Ocana-Gallegos, Edgar P. Spalding, Kevin M. Murphy, Amber L. Hauvermale

**Affiliations:** ^1^ Department of Crop and Soil Sciences, Washington State University, Pullman, WA, United States; ^2^ The Land Institute, Salina, KS, United States; ^3^ Department of Botany, University of Wisconsin, Madison, WI, United States

**Keywords:** *Chenopodium quinoa* Willd., preharvest sprout (PHS), seed dormancy, morphological traits, agronomic traits, phenetic modeling, high-throughput phenotyping related to the seed morphology and composition analysis

## Abstract

**Introduction:**

Quinoa is a high-value, nutritious crop that performs well in variable environments, marginal soils, and in diverse crop rotations. Quinoa’s many attributes make it an ideal crop for supporting human health in global communities and economies. To date, quinoa research has largely focused on traits in adult plants important for enhancing plant phenotypic plasticity, abiotic stress, disease resistance, and yield. Fewer studies have evaluated quinoa seed dormancy and suggest that most modern quinoa varieties have weak or no seed dormancy, and a narrow window of seed viability post-harvest. In other crops, diminished seed dormancy is a major risk factor for preharvest sprouting (PHS; germination on the panicle due to rain prior to harvest) and may also pose a similar risk for quinoa.

**Methods:**

This study (1) developed a dormancy screening assay to characterize seed dormancy strength in a large collection of quinoa varieties, (2) investigated if morphological variables including seed coat color, seed coat thickness, seed shape including eccentricity which evaluates the roundness or flatness of a seed, and other agronomic traits like crude protein content and seed moisture, contribute to quinoa seed dormancy, and (3) evaluated the use of a phenetic modeling approach to explore relationships between seed morphology and seed dormancy.

**Results:**

Dormancy screening indicated seed dormancy ranges in quinoa varieties from none to strong dormancy. Further, phenetic modeling approaches indicate that seed coat thickness and eccentricity are important morphological variables that impact quinoa seed dormancy strength.

**Conclusions:**

While dormancy screening and phenetic modeling approaches do not provide a direct solution to preventing PHS in quinoa, they do provide new tools for identifying dormant varieties as well as morphological variables contributing to seed dormancy.

## 1 Introduction

Quinoa (*Chenopodium quinoa* Willd.) is a popular food staple in households around the world and has immense potential to contribute to global food security (Murphy et al., 2016). Quinoa is nutrient dense and flavorful and is able to grow in a vast array of climates and conditions (Albani et al., 1997; Morales et al., 2017; Murphy et al., 2018). The wealth of genetic diversity in quinoa has also contributed to many of its appealing attributes including tolerance to abiotic stressors such as drought and salinity stress, making it a desirable crop for production in marginal growing areas (Jarvis et al., 2017; Hinojosa et al., 2018). However, quinoa was not originally adapted to the environmental conditions in the northern latitudes, or to large scale production resulting in unique challenges to breeders and farmers that seek to introduce it to new growing regions (Zurita-Silva et al., 2014; Peterson and Murphy, 2015; Hinojosa et al., 2018). Two emerging challenges facing quinoa production are preharvest sprouting (PHS) and loss of seed viability.

PHS is a phenomenon that results in mature seed germination on the mother plant. PHS is induced with rain prior to harvest and occurs as the result of insufficient or absence of seed dormancy (Benech-Arnold, 2001; Vetch et al., 2019). Seed dormancy prevents untimely germination, and lack of or weak seed dormancy is hypothesized to be a major risk factor for PHS in quinoa (Bewley, 1997; Bradford and Nonogaki, 2009; Arc et al., 2013; Willis et al., 2014). Dormancy classification takes into consideration the developmental state of the embryo at the time of seed dispersal, as well as physiological responses of the seed to environmental cues (Baskin and Baskin, 2004).

Primary dormancy is established during seed development, whereas secondary dormancy is the re-establishment of dormancy of mature seeds in specific environments (Bewley, 1997). Physiological dormancy, a type of primary dormancy, is set by the plant hormone abscisic acid (ABA) during embryo maturation (Nikolaeva, 1969; Bewley, 1997; Baskin and Baskin, 2004; McGinty et al., 2021). Seed germination is stimulated by the plant hormone gibberellin (GA). As seeds lose physiological dormancy though a period of dry storage (after-ripening) or with seed coat scarification, they become less responsive to ABA, and often display increased sensitivity to GA and increased GA signaling (Finch-Savage and Leubner-Metzger, 2006; Hauvermale et al., 2012; Bewley et al., 2013; Tuttle et al., 2015). Both primary dormancy loss and the absence of primary dormancy increase the likelihood of PHS with rain. However, the two physiological states are not equivalent with very different implications on breeding for PHS resistance. Quinoa PHS susceptibility may arise from the absence of primary dormancy or insufficient levels of primary dormancy at physiological maturity (McGinty et al., 2021). Taken together, this suggests that different quinoa varieties may have different dormancy states at maturity. Therefore, it is essential to determine if there are differences in quinoa dormancy strength, and if the apparent “absence” of quinoa dormancy stems from observable changes to primary dormancy beginning at physiological maturity, or if “absence” means that some quinoa varieties lack primary dormancy altogether.

The physical attributes of a seed, including seed coat thickness, color, and shape also contribute to seed dormancy physiology and strength in many crops and model species (Himi et al., 2002; Baskin and Baskin, 2004; Bradford and Nonogaki, 2009; Finkelstein et al., 2008; Tuttle et al., 2015; Tai et al., 2021). These same characteristics have been hypothesized to contribute to seed dormancy strength in quinoa as well. (Gil and Cubero, 1993; Himi et al., 2002; Ertekin and Kirdar, 2010; Ceccato et al., 2015; Williams, 2019; Tai et al., 2021). Ceccato et al. (2015) evaluated associations between dormancy strength, seed coat thickness, seed coat color, and retained endogenous ABA levels in two quinoa varieties, Chadmo and 2-Want. This study found that Chadmo, the variety with the thicker and darker seed coat, had stronger seed dormancy and higher retained endogenous levels of ABA, whereas 2-Want, the variety with the thinner and lighter seed coat, was less dormant and leached more ABA through the thinner seed coat. The result that quinoa seed coat color may influence dormancy strength, is consistent with studies that have found that wheat seed coat color is associated with differences in primary dormancy strength and located on the same quantitative trait loci (QTL) as PHS tolerance (Groos et al., 2002; Fofana et al., 2009; Lin et al., 2015; Vetch et al., 2019).

To investigate the physiological and physical attributes of quinoa seed dormancy, the goals of the current study were: (1) to use broadly used hormone screening methods (reviewed in Finch-Savage and Leubner-Metzger, 2006) and to develop dormancy ratings modeled after Tuttle et al. (2015) as a way to establish baseline parameters for classifying primary dormancy strength at physiological maturity under the described experimental conditions, (2) to investigate if morphological characteristics contribute to quinoa seed dormancy and might identify useful seed phenotypes for selection of dormant varieties, and (3) to build a broader understanding of primary dormancy mechanisms in quinoa, along with easy to use screening tools targeted to help breed for increased primary seed dormancy and eventually for PHS tolerance. To achieve these goals, a dormancy screening assay was developed to investigate the range of primary dormancy strength and/or types existing in a subset of a large, diverse panel of varieties from the quinoa world core collection (https://quinoa.kaust.edu.sa/#/data/germplasm; Patiranage et al., 2022; Craine et al., 2023). Additionally, a phenetic modeling approach, factorial analysis of mixed data (FAMD), was developed to evaluate associations between morphological characteristics, namely seed coat color and thickness and seed dormancy in quinoa. The FAMD approach was also expanded to evaluate the impacts of other important morphological and agronomic traits on dormancy, like crude protein, as these are routinely measured during selection and breeding. The list of traits included crude protein content, moisture content, area, solidity (seed convexity), perimeter, eccentricity (seed roundness or flatness), major axis length and minor axis length.

## 2 Materials and methods

### 2.1 Plant material, greenhouse conditions, and explanation of datasets

Varieties for this study included 189 varieties from the quinoa world core collection; seeds originated in Germany in the 2018 and 2019 growing seasons (Patiranage et al., 2022). Seeds were sown in 800 ml square pots filled with potting Mix (Sunshine Mix LC1 and LA4), and a ¼ teaspoon of Osmocote (Scotts’s 14-14-14 Classic). Fertilizer was added after thinning the plants. Plants were grown under the following greenhouse conditions for 5 months: 16 hours of light per day, a maximum daytime temperature of 23.8°C and 15.5°C at night. Panicles were harvested individually at physiological maturity, as defined by the Biologische Bundesanstalt Bundessortenamt und Chemische Industrie (BBCH)-scale (Sosa-Zuniga et al, 2017). Seeds were hand-threshed from panicles into separate paper coin envelopes and dried at room temperature under conditions with low relative humidity (15-30%) for seven days. Dry seeds were transferred to separate microfuge tubes stored in sealed plastic storage bins in the dark at -20°C under low moisture conditions to maintain seed moisture at > 10%. Previous studies in wheat, arabidopsis, and quinoa have demonstrated that storage at -20°C is useful for preserving dormancy status at harvest maturity for short storage durations, i.e., less than 6 months, and does not to negatively impact seed germinability (Mares, 1983; Romero et al., 2018; Nelson et al., 2023).

Of the varieties in the collection, dormancy was measured in all 189 (**Supplemental Table 1**). Seed coat thickness and seed coat color were measured for 181 varieties, and for a subset of 158 varieties, area, crude protein, eccentricity, major axis length, minor axis length, moisture, perimeter, and solidity were also measured (**Supplemental Table 2**). Finally, once seed viability was established, the subset of 158 varieties was used for FAMD analyses.

### 2.2 ABA dose-response and seed dormancy assays

Prior to beginning germination experiments, seeds that were stored at -20°C were allowed to warm to room temperature for 24 hours. Seeds were then surface sterilized for 5 minutes using 70% ethanol, rinsed with sterile water and then sown on sterile filter paper moistened with 4.5 ml of sterile water buffered with 5 mM 2-(N-morpholino) ethanesulfonic acid buffer (MES, pH 5.5) without hormone or containing 0.1 μM, 1 μM, or 10 μM ABA, and imbibed in the dark at room temperature (22°C) for efficient uptake of ABA (Schramm et al., 2010; Tuttle et al., 2015). For initial ABA-dose response measurements, each treatment was replicated three times, and each technical replicate contained ten seeds. Seed germination was visually scored after each day, and the average percentage germination was calculated for day seven for each variety. Seeds were initially categorized based on germinability in the absence of hormone and sensitivity to ABA. Seeds with germination percentages of 0-24% without treatment or in the presence of ABA were classified as having strong dormancy (SD). Seeds with less than 50% germination in the absence of hormone and decreasing rates of germination with increasing rates of ABA were classified as having dormancy (D). Seeds with greater than 50% germination in the absence of hormone but still responsive to ABA were classified as having weak dormancy (WD). Seeds that reached germination rates above 75% regardless of ABA concentration were classified as having no dormancy (ND).

To refine initial dormancy categories, a second round of plating assays was performed for the SD, D, and WD groups. In this case, germination assays were performed on three biological replicates, with three technical replicates for each treatment consisting of 10 seeds per replicate. Sterilized seeds were imbibed without hormone or in the presence of 10 μM ABA or 10 μM GA_3_ at room temperature (Schramm et al., 2010; Ariizumi et al., 2013; Hauvermale et al., 2015; Tuttle et al., 2015). A primary dormancy classification based on both ABA and GA hormone response (Tuttle et al., 2015) was assigned accordingly. Seeds displaying less than 25% germination without hormone or with ABA and less than 30% germination with GA were classified as having strong dormancy (SD). Seeds with 50% or greater germination on GA and less than 50% germination in the absence of hormone and decreasing germination rates with increasing concentrations of ABA were classified as dormant (D). Seeds with germination percentages above 50% without hormone or with GA but still responsive to ABA were classified as having weak dormancy (WD). Seeds initially categorized as ND were not retested with GA.

### 2.3 After-ripening and viability screening

Seeds classified as SD or D were after-ripened at room temperature for one month (AR). Unlike in Ceccato et al. (2015), which evaluated dormancy loss after 6 months of after-ripening, shorter times points were selected to capture subtle variations in dormancy loss that might occur in different quinoa varieties (Baskin and Baskin, 2004; Finch-Savage and Leubner-Metzger, 2006). AR seeds were surfaced sterilized and imbibed without hormone, or with 10 µM ABA or 10 GA_3_ µM as described above. Percent germination was determined on day 7 of imbibition. If AR seeds failed to germinate in the absence of ABA or with GA, viability was tested using scarification. Seeds to be scarified were surface sterilized and then the seeds coats were nicked with a pair of tweezers. Scarified seeds were then sown on water-moistened sterile filter paper but without hormone to determine viability (Ceccato et al., 2015). If after scarification, AR seeds failed to germinate in the absence of hormone, a subset of non-scarified seeds were allowed to after-ripen further for an additional 2 months for a total of 3 months of after-ripening (LAR) prior to replating. Hormone screens and scarification studies were performed as described above with three replicates of each treatment, and three technical replicates of ten seeds from three biological replicates. Analysis of variance (ANOVA) was used to identify statistically significant differences in percent germination with hormone treatment, after-ripening, and scarification. Germination data was arcsin transformed, due to the non-normality of the data, and then ANOVAs were performed on transformed data using the PROC GLM function and were compared using a Tukey’s all pairwise comparison in SAS version 9.4 (SAS Institute; Freeman and Tukey, 1950). For all experiments described *p* values ≤ 0.05 were considered significant.

### 2.4 Seed coat thickness

An electronic caliper (Mitutoyo 500-196-30 with 0.001 mm precision) was used to measure the thickness of seed coats removed from dry seeds with the aid of a scalpel and a standard dissecting microscope (Gil and Cubero, 1993; Ross et al., 2008; Hradilová et al., 2019). Prego et al. (1998) was used as a reference to accurately identify the anatomy of the seed and seed coat. The average seed coat thickness of ten randomly selected seeds was determined for each variety.

### 2.5 Seed coat color

Seed coat color was scored by eye with respect to standard red, green, and blue (RBG) and the 6^th^ edition of the Royal Horticulture Society (RHS) color sheet (Royal Horticultural Society, 1966) as previously described (Fao and Iniaf, 2013). The ascribed single-color identifiers were grouped to form categories. For example, if there were shades of colors within a sample or similar colors across samples such as beige, pale brown, wheat, and tan, they were all labeled beige.

### 2.6 Seed composition

Seed composition traits, and moisture content, were estimated using a PerkinElmer (formerly Perten) DA7250 Near-Infrared Spectrometer with a near-infrared (NIR) range of 950-1650 nm and absorbance values recorded at every 5 nm (PerkinElmer, Waltham, MA, USA). Approximately 5-10 g of whole, unprocessed seed samples were analyzed according to Craine et al., 2023. For each predicted parameter, samples were excluded if they had predicted values outside the calibration range provided according to Stanschewski et al (2021). A second filtering step excluded samples if the Mahalanobis distances significantly differed (*p* < 0.001) from the spectra belonging to samples in the calibration data set. Mahalanobis distances for the experimental samples were determined using a covariance matrix calculated from the raw spectra values belonging to both the experimental and calibration samples, and a centroid calculated using raw spectra values from the calibration samples. A χ^2^ distribution with degrees of freedom equal to the total number of measured wavelengths in the NIR range (*n* = 141) and alpha equal to 0.001 were used to calculate *p* values. These two filtering steps were used to exclude samples from the data set, before raw predicted values were adjusted to dry matter content.

### 2.7 Seed morphology

An 8-bit red, green, blue (RGB) image of 1-2 g of seeds scattered on the bed of a document scanner (Epson Perfection V39, Epson America, Inc., Las Alimitos, CA) was collected for each sample at a resolution of 1200 dots-per-inch. The AllGrains custom image analysis software in the phytoMorph Image Phenomics Toolkit available in the Cyverse Discovery Environment (http://de.cyverse.org) extracted seed features from each image. The AllGrains tool also counted each seed in each image, including those touching each other in clusters, using the approach developed for counting maize kernels in similar images (Miller et al., 2017). The AllGrains tool determined the average seed area, perimeter, major and minor axis lengths, and their ratio (eccentricity) using an approach developed for Arabidopsis seeds (Moore et al., 2013). AllGrains automatically produced a mask image that the user could inspect to assess the quality of the image processing of each sample.

### 2.8 Factorial analysis of mixed data

FAMD analysis is a phenetic modeling approach that is a type of principal component analysis (PCA) and multiple correspondence analysis (MCA) (Pages, 2004). FAMD analysis is unique as it combines both quantitative and qualitative variables making it possible to explore the association between individuals by considering all mixed variables (Kassambara, 2017). Quinoa morphological data and dormancy categories were analyzed using FAMD analysis (Husson et al., 2007; Josse and Husson, 2016). In total four FAMD analyses were performed to identify global and specific variables associated with seed dormancy. In the first analysis all morphological and agronomic variables described above in sections 2.4 – 2.7 were compared with seed dormancy. In the second analysis only major seed characteristics with previously described connections with seed dormancy in quinoa, i.e. coat color and seed coat thickness, were used (Ceccato et al., 2015). In the third analysis, seed shape characteristics including area, eccentricity, major and axis length, perimeter, and solidity were compared with seed dormancy. Finally, in the fourth analysis, only seed moisture and crude protein were compared with seed dormancy. In all analyses 158 quinoa varieties were used, and the dimensions selected explained greater than 50% of the cumulative variance (Husson et al., 2007; Kassambara, 2017). Analyses were conducted in the R-Studio environment (R version 4.0.2 (www.r-project.org) using the packages factoextra, factoMineR, and FactoShiny (Husson et al., 2007; Isard et al., 2007; Lê et al., 2008; Vaissie et al., 2015; Husson et al., 2016; Josse and Husson, 2016; Kassambara and Mundt, 2017; Sievert, 2018).

### 2.9 Ordinal logistic regression models

OLRs (Agresti, 2002) are used when response variables are a mixture of categorical and continuous variables, which are ordered. In the case of this study, dormancy category variables (ND, WD, D, SD) were treated as categorical and ordinal, and analyzed relative to the predictor variables which were either categorical or continuous. Seed coat color and dormancy strength were treated as categorical variables, and area, seed coat thickness, crude protein, eccentricity, major axis length, minor axis length, moisture, perimeter, and solidity, were treated as continuous variables. The color beige was used as the baseline in the OLR model evaluating the impact of seed coat color, because it was the most prevalent color among all varieties evaluated. Because dormancy ranged from non-dormant (ND) to strongly dormant (SD) dormancy categories were defined as (1) ND, (2) WD, (3) D, and (4) SD. Analyses were conducted in the R-Studio environment (R version 4.0.2 (www.r-project.org) using the package MASS (Venables and Ripley, 2002), and *p* values of ≤0.05 were considered statistically significant.

## 3 Results

### 3.1 Evaluating dormancy at physiological maturity

**Figure 1:**
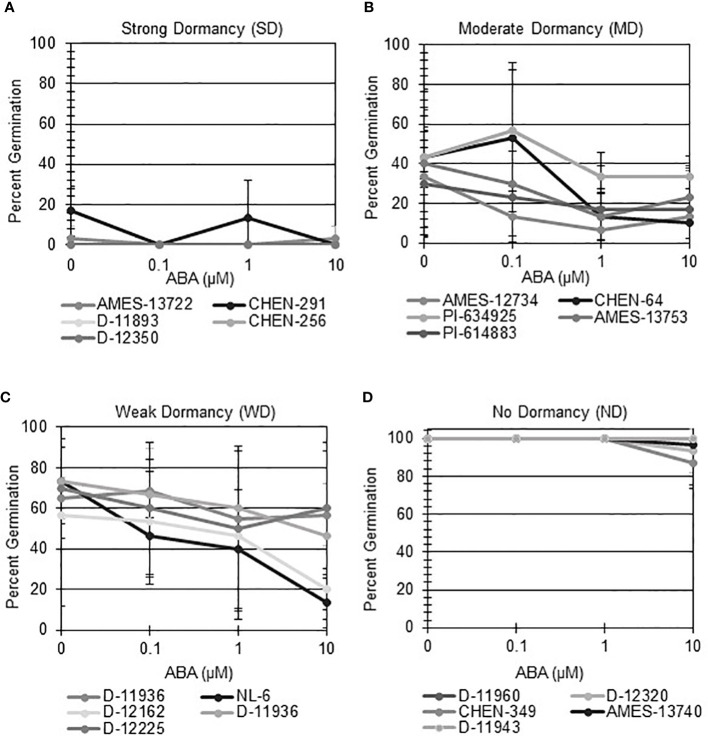
Seed from different quinoa varieties display a range of sensitivity to ABA at physiological maturity. ABA-does response curves were performed to evaluate if different quinoa varieties display varying degrees of seed dormancy at physiological maturity. All varieties were imbibed at 22°C in the dark for 7 days in the absence of ABA (water buffered with 5 mM MES), and in the presence of 0.1 µM, 1 µM, and 10 µM ABA. A preliminary dormancy category was assigned based on percent germination across all treatments on day 7. A sub-set of 5 varieties from each category was selected to illustrate dormancy assignment. **(A)** Varieties with strong dormancy (SD) germinated at < 25% in the presence or absence of ABA. **(B)** Varieties were classified as having moderate dormancy (MD) when germination rates were < 50% in the absence of ABA and decreased with increasing concentrations of ABA. **(C)** Varieties with weak dormancy (WD) had germination rates > than 50% in the absence of ABA, but which decreased with increasing concentrations of ABA. **(D)** Varieties with no apparent dormancy (ND) germinated at > 95% in the absence of hormone and reached germination rates > 75% in the presence of 10 µM ABA. Percent germination was calculated as the mean of three biological replicates with 10 seeds each. Error bars represent standard deviation.

Seeds that display strong primary dormancy do not germinate when imbibed in favorable conditions. Moreover, seeds with differences in primary dormancy at physiological maturity retain different degrees of sensitivity to ABA and GA. To determine if quinoa varieties displayed differences in primary dormancy, specifically physiological dormancy, at physiological maturity, seeds from a total of 189 varieties were first imbibed without hormone, or across an ABA gradient (Bewley, 1997). Initial dormancy category assignments were modeled after those previously defined by Tuttle et al. (2015) for wheat (**Figure 1**; **Supplemental Table 1**). Quinoa varieties with strong dormancy (SD) germinated at < 25% in the presence or absence of ABA (**Figure 1A**). Varieties with moderate dormancy (MD) germinated at < 50% in the absence of ABA and germinated less in the presence of increasing concentrations of ABA (**Figure 1B**). Varieties with weak dormancy (WD) germinated at > than 50% in the absence of ABA, but still responded to increasing concentrations of ABA (**Figure 1C**). Varieties without apparent dormancy (ND) germinated at > 95% in the absence of hormone and reached germination rates > 75% in the presence of 10 µM ABA (**Figure 1D**). A total of 12 varieties were classified as SD, 9 were classified as MD, 27 were classified as WD, and 141 were classified as ND.

**Figure 2:**
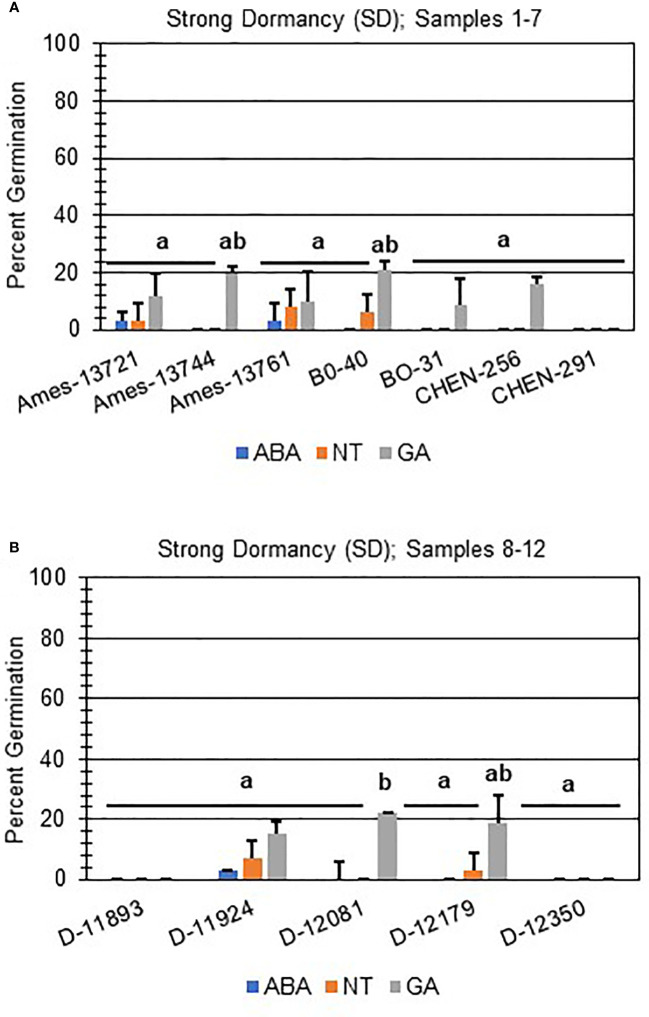
Hormone screens confirm quinoa varieties with strong primary dormancy at physiological maturity. Hormone screens were performed on 12 quinoa varieties, **(A)** samples 1-7 and **(B)** 8-12, with little germination in the absence of hormone and 10 µM ABA at physiological maturity. All seeds were imbibed on filter paper in the dark for 7 days at 22ºC without hormone (NT; water buffered with 5 mM MES), or in the presence of 10 µM ABA or 10 µM GA. Varieties with strong dormancy (SD) germinated at < 25% in the presence or absence of ABA, and < 30% in the presence of GA. Percent germination for all treatments was the average of three technical replicates, with 10 seeds each, from three biological replicates. An acrsin transformation was used to normalize germination data, and letters indicate statistically different categories (p value < 0.05) based on ANOVA with a Tukey’s pairwise comparison. Error bars represent standard deviation.

**Figure 3:**
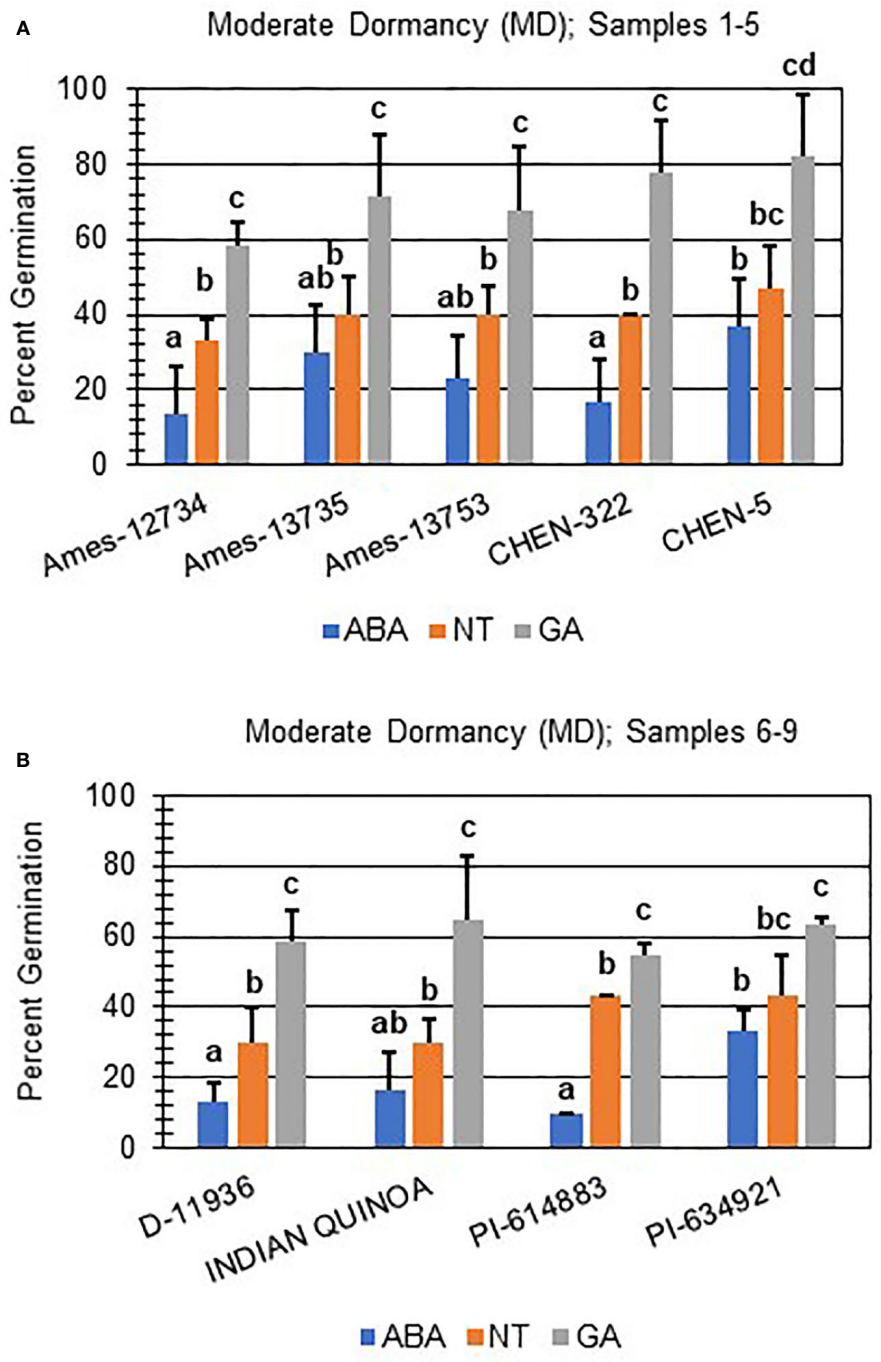
Hormone screens confirm quinoa varieties with moderate primary dormancy at physiological maturity. Hormone screens were performed on 9 quinoa varieties, **(A)** samples 1-5 and **(B)** 6-9, some germination in the absence of hormone and response to 10 µM ABA at physiological maturity. All seeds were imbibed on filter paper in the dark for 7 days at 22ºC without hormone (NT; water buffered with 5 mM MES), or in the presence of 10 µM ABA or 10 µM GA. Varieties with moderate dormancy (MD) germinated at > 50% with GA, and < 50% without hormone or ABA. Percent germination for all treatments was the average of three technical replicates, with 10 seeds each, from three biological replicates. An acrsin transformation was used to normalize germination data, and letters indicate statistically different categories (p value < 0.05) based on ANOVA with a Tukey’s pairwise comparison. Error bars represent standard deviation.

**Figure 4:**
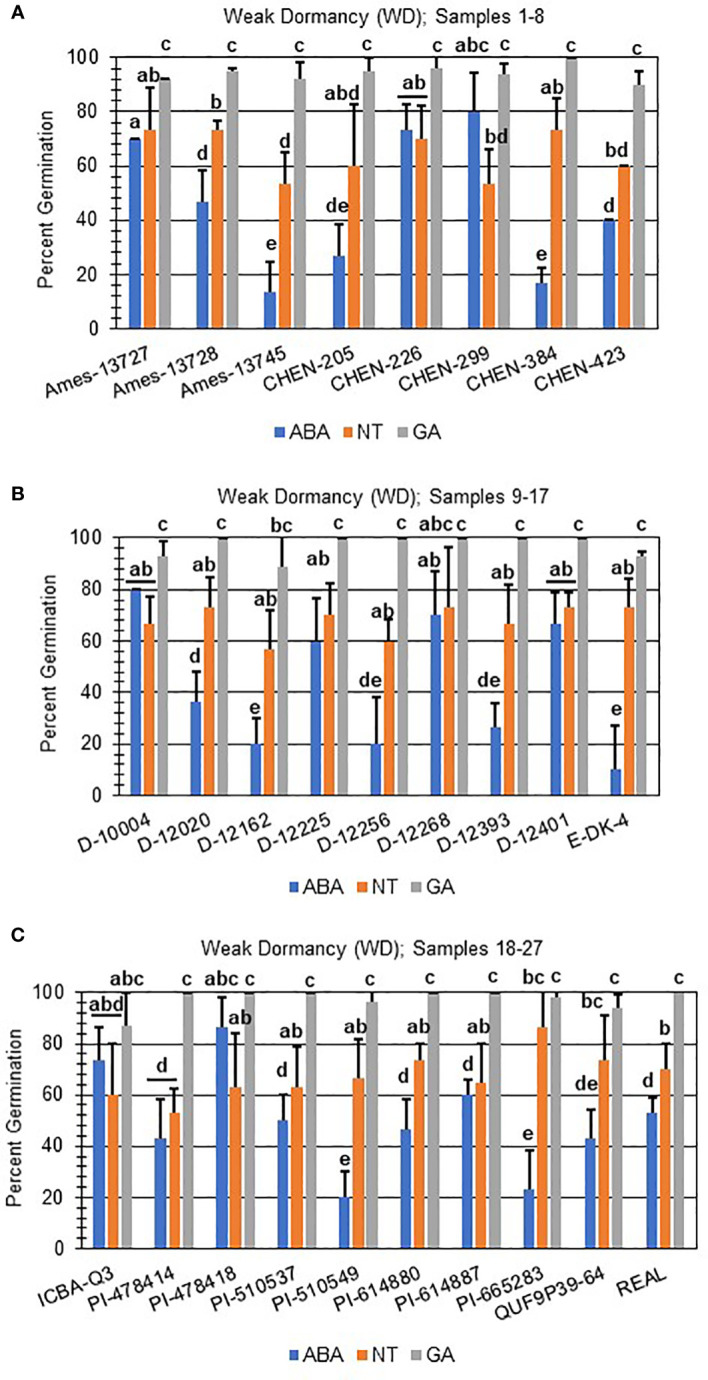
Hormone screens confirm quinoa varieties with weak primary dormancy at physiological maturity. Hormone screens were performed on 27 quinoa varieties, **(A)** samples 1-8, **(B)** 9-17, and **(C)** 18-27 with high rates of germination without hormone but still responsive to 10 µM ABA at physiological maturity. All seeds were imbibed on filter paper in the dark for 7 days at 22ºC without hormone (NT; water buffered with 5 mM MES), or in the presence of 10 µM ABA or 10 µM GA. Varieties with weak dormancy (WD) germinated at > 50% without hormone or GA, and with decreased sensitivity to ABA. Percent germination for all treatments was the average of three technical replicates, with 10 seeds each, from three biological replicates. An acrsin transformation was used to normalize germination data, and letters indicate statistically different categories (p value < 0.05) based on ANOVA with a Tukey’s pairwise comparison. Error bars represent standard deviation.

Next, hormone screening comparing differences in percent germination with seed imbibition with no treatment (NT), with 10 µM ABA, and 10 µM GA was used to establish a baseline for dormancy type and strength under controlled conditions for all varieties initially labeled SD, MD, or WD as a way to better define initial dormancy categories. Hormone screens confirmed initial dormancy classifications and that primary (physiological) dormancy ranged from strong (SD) to weak (WD) in 48 varieties (**Figure 1**). As before, germination percentages for the twelve SD varieties were < 25% in the absence of ABA, and even lower, and in many instances zero, in presence of 10 µM ABA. Additionally, these varieties displayed a high degree of GA-insensitivity, with germination rates less than 30% (**Figure 2**). Further, there were no significant differences between ABA and NT, and with the exception of D-12081, there were no significant differences between NT and GA treatment. Of the 9 varieties initially categorized as MD, all displayed a significant response to GA compared to NT and ABA treatments at physiological maturity reaching > 50% germination (**Figure 3**; *p* values 0.05). Germination percentages for these varieties was < 50% without GA and all MD varieties retained ABA-sensitivity. All varieties initially labeled as WD, reached > 75% germination with GA, and > 50% germination with NT (**Figure 4**). WD varieties also showed increased ABA-insensitivity, with some including D-10004 and PI-478418 displaying similar rates of germination with NT or GA treatment (**Figures 4B, C**).

### 3.2 After-ripening and viability screening

**Figure 5:**
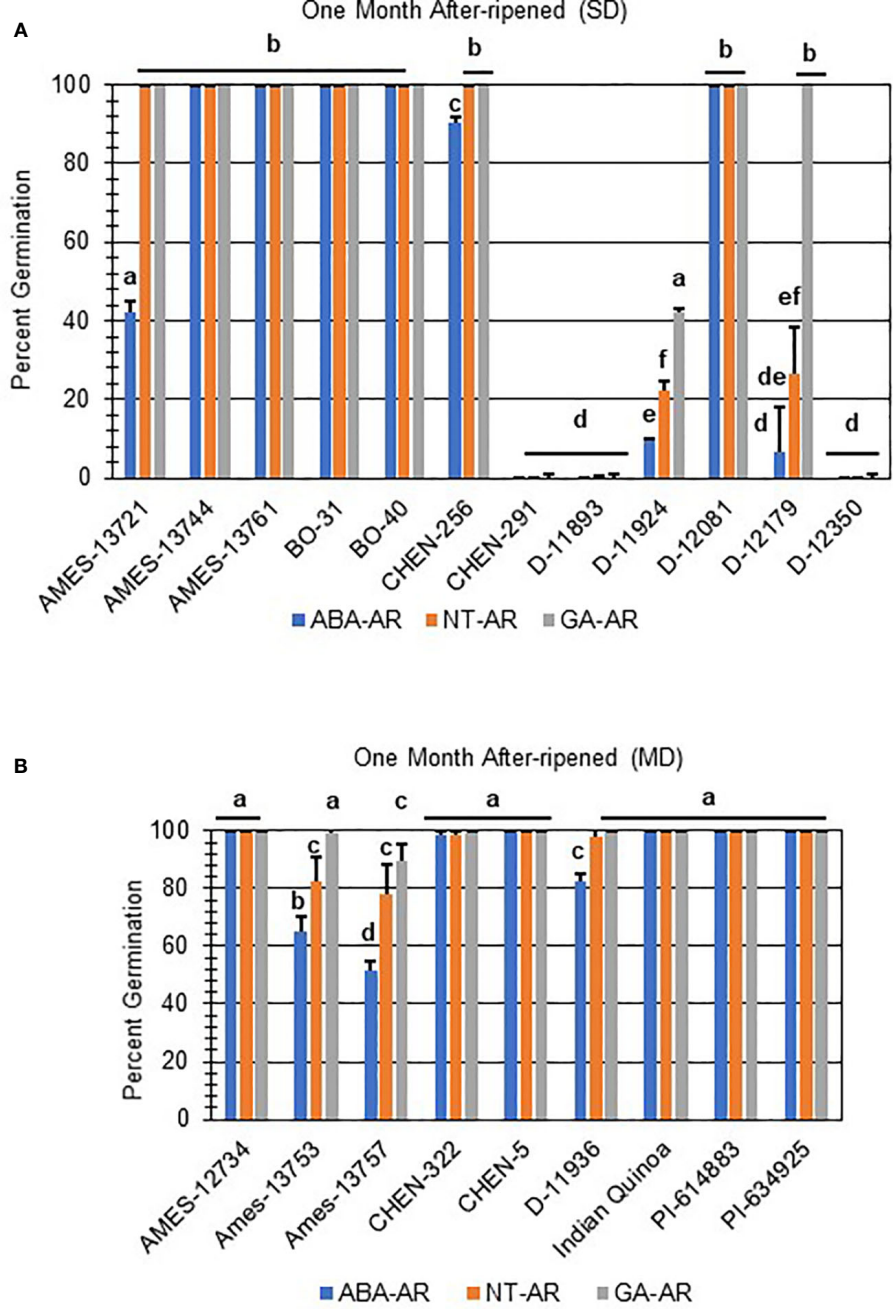
After-ripening improved seed germination in some quinoa varieties with strong and moderate seed dormancy at physiological maturity. **(A)** SD and **(B)** MD quinoa varieties were after-ripened for one month at 22°C. After-ripened seeds were imbibed on filter paper in the dark for 7 days at 22°C without hormone (NT; water buffered with 5 mM MES), or in the presence of 10 µM ABA or 10 µM GA. Percent germination for all treatments was the average of three technical replicates, with 10 seeds each, from three biological replicates. An acrsin transformation was used to normalize germination data, and letters indicate statistically different categories (p value < 0.05) based on ANOVA with a Tukey’s pairwise comparison. Error bars represent standard deviation.

Both strong primary dormancy and issues with seed viability cause poor seed germination at physiological maturity. After-ripening, a period of dry storage, is one treatment that breaks dormancy in orthodox seeds and results in increased seed germination (Koornneef and Karssen, 1994). Other dormancy breaking treatments include scarification and cold imbibition (Koornneef and Karssen, 1994). To determine if the poor germination in SD- and MD-labeled varieties was due to dormancy or resulted from poor seed viability, all were after-ripened for one month and reevaluated for germination potential (**Figure 5**). A total of 8 of 12 after-ripened SD samples (**Figure 5A**), and all of the MD (**Figure 5B**) samples reached > 75% germination with GA imbibition and without hormone. Additionally, 5 after-ripened SD samples, and 7 MD samples lost all sensitivity to ABA reaching 100% germination regardless of treatment.

Two of the after-ripened varieties, D-11924 and D-12179, had germination rates < 50% without treatment, and showed a significant response to ABA compared to NT and GA treatment (**Figure 5A**; *p* values ≤ 0.05). Both also showed an increase in seed germination compared to the dormant seeds (p-value ≤ 0.05). Interestingly, after-ripened D-12179 was more responsive to GA than D-11924, although both showed comparable rates of germination without hormone and in the presence of ABA. Taken together, these results suggest that D-12179 might reach complete dormancy loss faster than D-11924. Finally, after-ripened SD varieties CHEN-291, D-11893, and D-12350 showed no significant differences when compared with non-after-ripened SD varieties regardless of treatment. These varieties also displayed GA-insensitivity and significantly reduced germination compared to all other one month after-ripened SD or MD varieties (**Figure 5**; *p* values ≤ 0.05).

**Figure 6:**
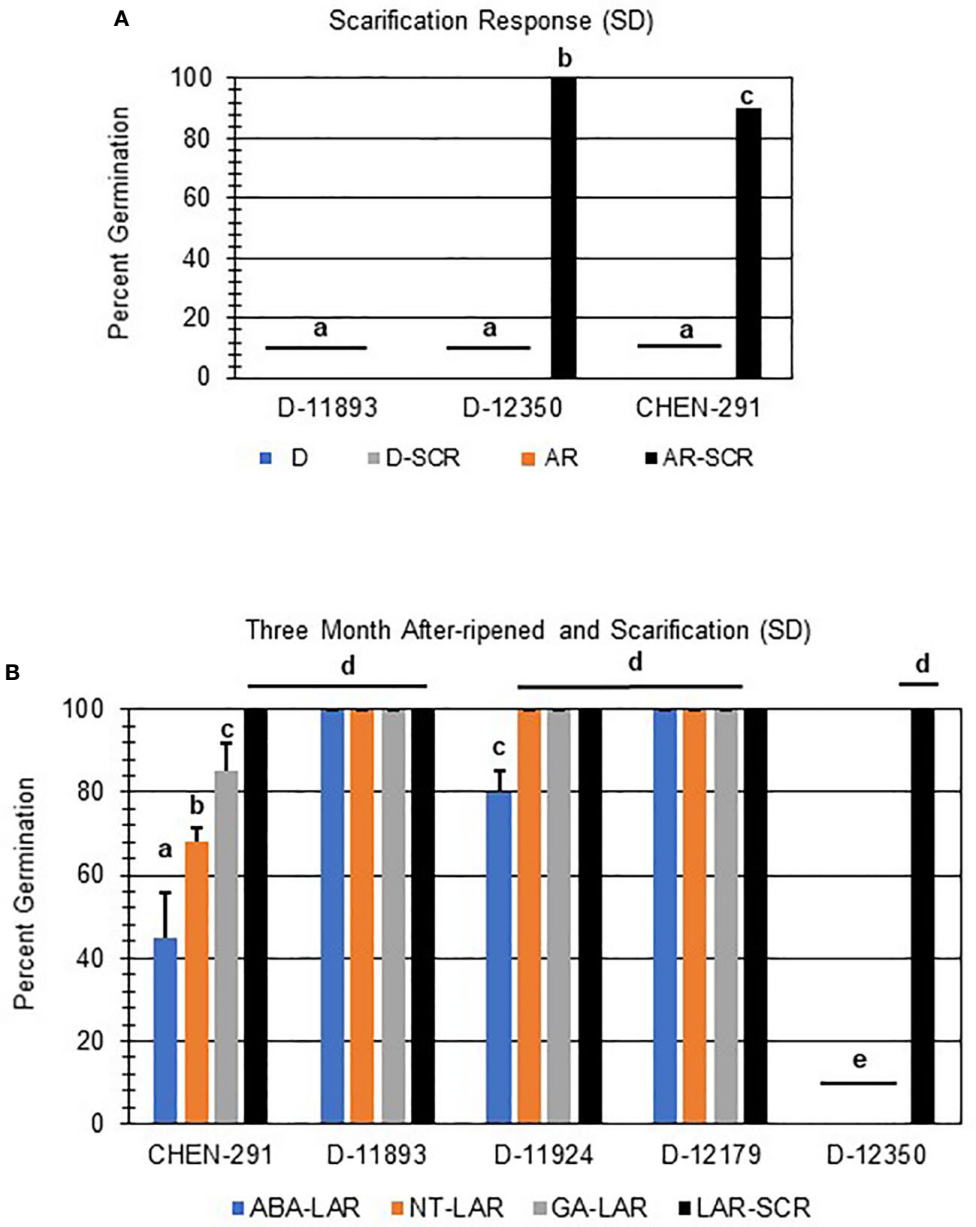
Scarification and long after-ripening improved seed germination in quinoa varieties with strong primary dormancy at physiological maturity. **(A)** SD quinoa varieties with low rates of germination at physiological maturity and one month of after-ripening (AR) were scarified (SCR) and then imbibed without hormone in the dark for 7 days at 22ºC. **(B)** Quinoa varieties displaying low rates of germination after 1 moth of after-ripening were further after-ripened for a total of 3 months (LAR). LAR seeds were scarified or plated directly and imbibed on filter paper in the dark for 7 days at 22ºC without hormone (NT; water buffered with 5 mM MES), or in the presence of 10 µM ABA or 10 µM GA. Percent germination for all treatments was the average of three technical replicates, with 10 seeds each, from three biological replicates. An acrsin transformation was used to normalize germination data, and letters indicate statistically different categories (p value < 0.05) based on ANOVA with a Tukey’s pairwise comparison. Error bars represent standard deviation.

Further evaluation of viability of non-germinating or poorly germinating varieties using scarification indicated that D-12350 and CHEN-291 were viable and showed significant increases in germination (**Figure 6A**; p-value ≤ 0.05). This result suggests that these varieties may have seed coat-imposed dormancy. Interestingly, scarification and one month of after-ripening did not rescue D-11893 (**Figure 6A**), suggesting initially that D-11893 may have viability issues. However, when the same varieties, CHEN-291, D-11892, D-11924, D-12179, and D-12350, were allowed to after-ripen further for a total of three months, only D-12350 remained insensitive to all dormancy treatments except for scarification (**Figure 6B**). Taken together these results suggest that: (1) at physiological maturity D-11893 has embryo-imposed dormancy and requires a longer period of after-ripening to achieve maximum germination potential compared to the other varieties tested, (2) D-12350 has seed coat-imposed dormancy and requires scarification for dormancy release, and (3) all varieties tested were viable at harvest.

### 3.3 Seed coat color, thickness, and morphology

**Figure 7:**
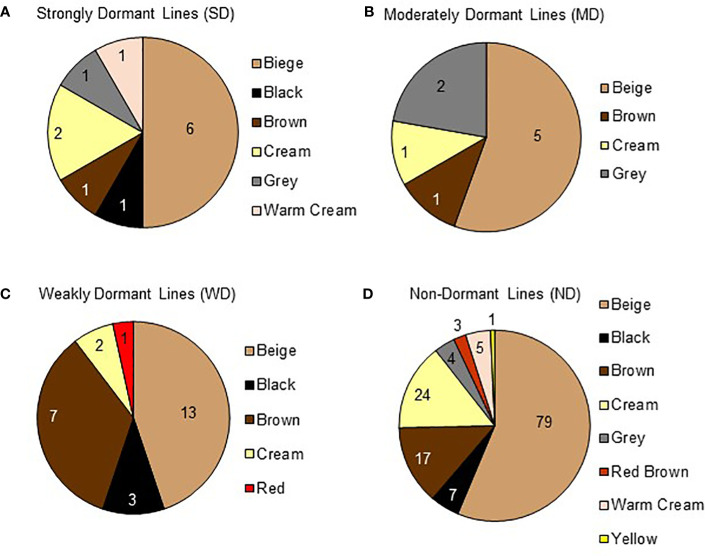
Quinoa seed coat color varies within dormancy categories. Seed coat color for 181 varieties was documented at the beginning of dormancy screening when seeds were at physiological maturity. Pie charts show the total number of varieties in each dormancy category, and each variety's seed coat color. Moving clockwise, each pie chart is ordered from most prevalent to least prevalent seed coat color. **(A)** A total of 12 varieties displayed strong dormancy (SD) and fell into 6 seed coat color categories; 6 beige, 1 black, 1 brown, 2 cream, 1 grey, and 1 warm cream. **(B)** A total of 9 varieties were we moderately dormant (MD) and fell into 4 seed coat color categories; 5 beige, 1 brown, 1 cream, and 2 grey. **(C)** A total of 26 varieties displayed weak dormancy (WD) and fell into 5 seed coat color categories; 13 beige, 3 black, 7 brown, 2 cream, and 1 red. **(D)** A total of 137 varieties appeared to have no dormancy (ND) and fell into 8 seed coat color categories; 79 beige, 7 black, 17 brown, 24 cream, 4 grey, 3 red brown, 5 warm cream, and 1 yellow.

Seed coat color and seed coat thickness were evaluated in 181 varieties. Seed coat color was grouped according to dormancy strength at physiological maturity (**Figure 7**). A total of nine colors were identified and there was no apparent association between seed coat color and dormancy strength. Beige was the most prevalent color and observed in 104 varieties. Cream was the second most abundant color and observed in 29 varieties. Brown was the third most abundant color recorder and observed in 26 varieties. Beige, cream, or brown colored seeds fell into all of the identified dormancy strength categories. Less common seed coat colors observed were black, grey, red, red brown, and yellow. Of those, the only seed coat colors associated with a single dormancy category at physiological maturity were red (WD), red brown (ND), and yellow (ND). Collectively, these observations suggest that within the subset of the quinoa world collection tested, seed coat color may not be a good predictor of seed dormancy.

In previous studies seed morphological variables have been suggested to contribute to seed dormancy and germination physiology in maize, weeds, and in quinoa (Balkaya and Odabas, 2002; Ceccato et al., 2015; Cervantes et al., 2016). In the current study, direct comparisons of dormancy category and the averages for morphological variable measurements, area, seed coat thickness crude protein content and moisture content, eccentricity, major and minor axis length, moisture, and perimeter, revealed no apparent segregation according to dormancy type (**Table 1**; **Supplemental Table 2**). It was noted however, the greatest seed coat thicknesses, and smallest ranges of eccentricity were recorded among the SD- and MD-varieties. For example, SD variety D-11924 had the thickest seed coat among all varieties tested (**Supplemental Table 2**).

**Table 1:** Averages and ranges for morphological variable according to dormancy category.

Secondary Morphological Variables		Dormancy Types			
		ND	WD	MD	SD
Area	Average	2.35	2.03	1.93	2.28
	Range	0.43 – 3.24	0.31 – 3.16	1.20 – 2.30	1.87- 2.86
Protein	Average	15.62	15.67	15.55	15.70
	Range	8.355 - 20.08	12.70 - 18.01	13.21 - 17.18	13.45 - 17.99
Thickness	Average Range	0.04 0.02 - 0.08	0.05 0.04 - 0.06	0.05 0.03 – 0.10	0.05 0.02 -0.12
Eccentricity	Average	0.39	0.44	0.40	0.40
	Range	0.34 - 0.84	0.34 - 0.85	0.34 - 0.68	0.36 -0.49
Major Axis	Average	1.81	1.71	1.68	1.80
	Range	1.18 – 2.16	1.22 – 2.11	1.37 – 1.80	1.60 – 2.04
Minor Axis	Average	1.64	1.50	1.47	1.62
	Range	0.59- 1.99	0.60 – 1.93	1.04 – 1.65	1.48 – 1.81
Moisture	Average	8.14	8.28	7.89	8.10
	Range	7.67 - 8.41	7.81 - 9.09	7.37 - 8.35	7.69 - 8.39
Perimeter	Average	6.05	6.02	5.97	6.27
	Range	4.48 – 7.46	4.29-7.75	5.21- 7.52	4.98 – 8.93

Area is reported in mm^2^; crude protein (Protein) and moisture are reported as grams per 100 g sample; seed thickness, major and minor axis, and perimeter are reported in mm.

### 3.4 Factorial analysis of mixed data

Four FAMD analyses were performed to evaluate relationships between morphological and agronomic variables and seed dormancy in quinoa. If the variables tested impacted dormancy strength, the expectation would be to see clustering based on the previously defined dormancy strength categories (SD, MD, WD, and ND). A combination of clustering and dimensional percentages was used to evaluate accuracy of the analysis. Percentages of inertia or dimensional percentages that were below 50% when combined for each dimension were not considered significant or to support the FAMD analysis. However, when the dimensional percentages were approximately 50% or greater this suggested that there was the likelihood of a correlation between the clustered variables (Pages, 2004; Pages, 2014; Husson et al., 2016; Kassambara, 2017).

**Figure 8:**
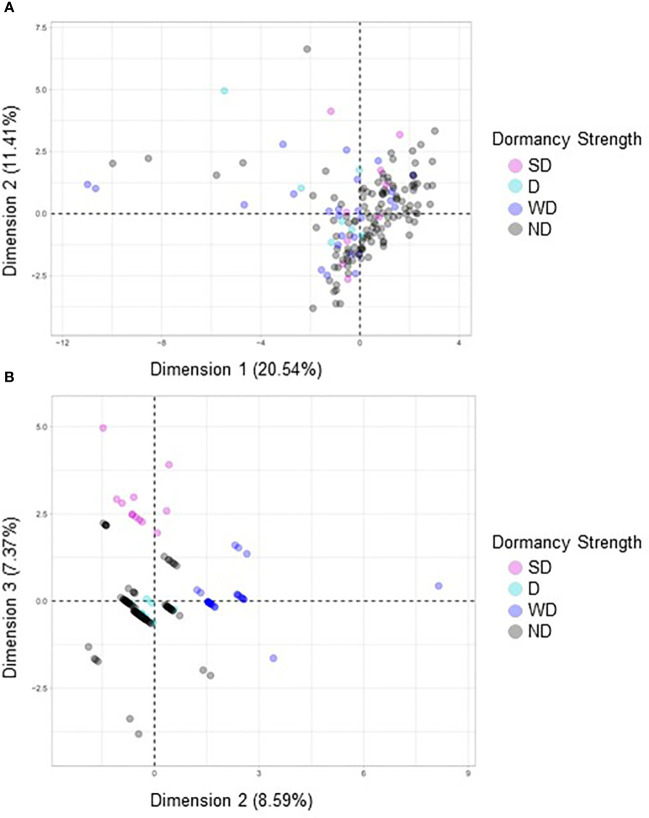
A factorial analysis of mixed data (FAMD) was performed with a broad set of morphological and quality traits to identify associations with dormancy strength. **(A)** In the first model variables compared included area, seed coat color, seed coat thickness, crude protein, eccentricity, major axis length, minor axis length, moisture, perimeter, solidity, and dormancy strength. **(B)** In the second model only seed coat color, seed coat thickness and dormancy strength were compared. For each model 158 varieties were compared, and each point represents a unique variety.

**Figure 9:**
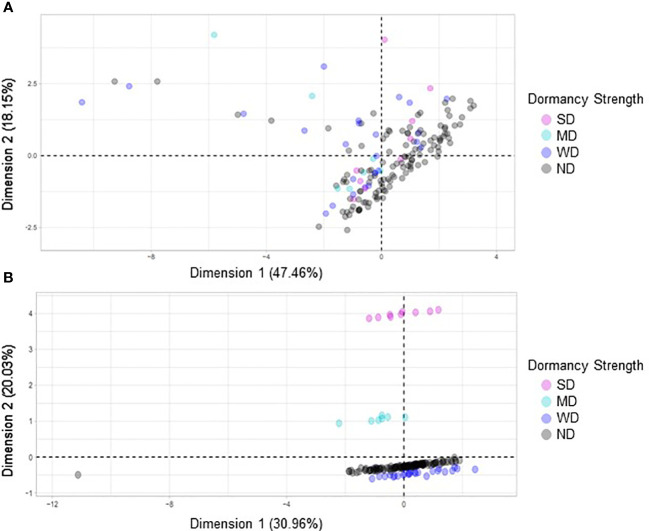
A factorial analysis of mixed data (FAMD) was performed using a subset of secondary morphological variables and quality traits to identify associations with dormancy strength. **(A)** In the first model variables compared included area, eccentricity, major axis length, minor axis length, perimeter, solidity, and dormancy strength. **(B)** In the second model crude protein, moisture content, and dormancy strength were compared. For each model 158 varieties were compared, and each point represents a unique variety.

FAMD analyses were performed to evaluate possible correlations between seed dormancy and morphological variables including area, seed coat color, seed coat thickness, eccentricity, major axis length, minor axis length, perimeter, solidity, crude protein, and seed moisture (**Figure 8**). When all variables were compared no observable correlations were seen (**Figure 8A**). However, when seed coat thickness and color were evaluated with dormancy category, clustering was observed (**Figure 8B**). Separate FAMD analyses were also performed to determine if in the absence of seed coat color and thickness, other seed morphological variables, area, eccentricity, major axis length, minor axis length, perimeter, solidity, or the physiological variables crude protein and moisture predict seed dormancy (**Figure 9**). Although, no distinct clustering with dormancy wast noted, higher dimensional percentages were achieved (**Figure 9A**). Moreover, when crude protein and seed moisture were isolated from other secondary morphological variables, stronger correlations were observed (**Figure 9B**).

### 3.5 Ordinal logistic model

OLR was used to determine the statistical significance of correlations between morphological and physiological variables, and dormancy strength observed with FAMD. OLR analysis was used for both single and combined variables. In total, three analyses were performed to evaluate correlations between morphological, physiological, and dormancy components of quinoa. In the first analysis evaluating seed coat thickness and color, only seed coat thickness showed a significant categorical effect on dormancy, and also significant differences between dormancy categories (*p* values < 0.005; **Table 2**). This result is not surprising, given there was no observable segregation of seed coat colors into a specific dormancy category (**Figure 7**; **Table 2**). OLR analysis performed by comparing protein and moisture content with dormancy strength indicated that neither variable had a significant effect, in combination or alone, on seed dormancy strength (**Table 3**). Lastly, combinatorial analysis of seed area, solidity, perimeter, eccentricity, and major and minor axis length indicated that these traits appeared to have some significant effects on seed dormancy (*p* values < 0.005; **Table 4**). However, when components were compared individually with dormancy, only eccentricity had a significant impact on seed dormancy (*p* values < 0.005; **Table 4**).

**Table 2:** Ordinal logistic model for prediction of dormancy, given seed coat color and thickness.

Ordinal Logistic Model				
Variables	Estimate	Standard Error	t-value	p-value
Seed Coat Thickness	22.31746770	11.3523844	1.96588372	0.050
Black	0.58857742	0.6558007	0.89749433	0.370
Brown	0.54717835	0.4486604	1.21958234	0.220
Cream	-0.28399637	0.5484594	-0.51780744	0.600
Grey	0.39675896	0.6704292	0.59179847	0.550
Red	-0.01308446	1.1532059	-0.01134616	0.990
Yellow	-0.60221553	1.1191818	-0.53808554	0.590
1|2	2.17390433	0.5855012	3.71289467	0.001
2|3	3.27168992	0.6249576	5.2350581	0.001
3|4	3.89583622	0.6604478	5.89878030	0.001

The dormancy response variables are 1= no dormancy (ND), 2 = weak dormancy (WD), 3 = moderate dormancy (MD), 4= strong dormancy (SD). Dormancy variables were modeled after those in Tuttle et al. (2015), and boundaries between categories are indicated with brackets. *P* values of ≤ 0.05 are considered significant.

**Table 3:** Ordinal logistic model for prediction of dormancy, given crude protein and moisture.

Ordinal Logistic Model				
Variables	Estimate	Standard Error	t-value	p-value
Crude Protein	0.00998866	0.1291382	0.07734860	0.940
Moisture	0.01833206	0.3339278	0.05489828	0.960
1|2	1.4126731	2.4006179	0.58846195	0.560
2|3	2.53730737	2.4078088	1.05378276	0.290
3|4	3.15845931	2.4161570	1.30722435	0.190

The dormancy response variables are 1= no dormancy (ND), 2 = weak dormancy (WD), 3= moderate dormancy (MD), 4= strong dormancy (SD). Dormancy variables were modeled after those in Tuttle et al. (2015), and boundaries between categories are indicated with brackets. *P* values of ≤ 0.05 are considered significant.

**Table 4:** Ordinal logistic model for prediction of dormancy, given area, solidity, perimeter, and eccentricity of the seed.

Ordinal Logistic Model				
Variables	Estimate	Standard Error	t-value	p-value
Area	-0.01317454	0.010819811	-1.2176308	0.220
Solidity	1.85781191	1.438185049	1.2917753	0.200
Perimeter	0.01043572	0.008211788	1.2711868	0.200
Eccentricity	6.73418626	1.249065727	5.3913786	0.001
Major Axis	-0.09584285	0.113080391	-0.8066223	0.420
Minor Axis	0.09584285	0.055901986	1.7144803	0.090
1|2	7.12908792	2.090581815	3.4100975	0.001
2|3	8.33195883	2.105977441	3.9563381	0.001
3|4	8.97111514	2.121974973	4.2277196	0.001

Dormancy response variables are 1= no dormancy (ND), 2 = weak dormancy (WD), 3 = dormancy (MD), 4= strong dormancy (SD). Dormancy variables were modeled after those in Tuttle et al. (2015), and boundaries between categories are indicated with brackets. *P* values of ≤ 0.05 are considered significant.

## 4 Discussion

### 4.1 Seed dormancy in quinoa

Insufficient seed dormancy in quinoa is thought to be a result of the broader introduction of this crop into geographic areas outside of its region of origin (Ceccato et al., 2011; Ceccato et al., 2015). Reduced dormancy in quinoa is also likely the consequence of cultivation and selection of favorable agronomic traits such as emergence, seed size, and seed coat thickness (Bruno, 2006; Ceccato et al., 2015; Hauvermale and Sanad, 2019). However, variation in dormancy testing regimes and limited variety testing in previous studies have made a broader understanding of seed dormancy in quinoa difficult. Observations from previous studies indicate that some quinoa varieties have primary seed dormancy at physiological maturity while others appear to have no primary dormancy at all (Farnsworth, 2000; Ceccato et al., 2015). In general, dormant seeds respond to ABA and are GA-insensitive. As viable seeds lose dormancy, they become ABA-insensitive and rates of germination improve as GA signaling pathways are turned on (Koornneef and Karssen, 1994; Bewley, 1997; Benech-Arnold, 2004; Finch-Savage and Leubner-Metzger, 2006). In seeds with viability issues, germination rates do not improve. In orthodox seeds, and certain quinoa varieties, which possess seed dormancy and are desiccation tolerant, issues with viability associated with storage may arise from seed protein insolubility (Castellion et al., 2010). Issues with viability sometimes associated with the absence of a primary dormancy program, and observed with some quinoa varieties, may also indicate that some quinoa varieties may have an unorthodox, or recalcitrant seed developmental program (Farnsworth, 2000). Recalcitrant seeds do not have seed dormancy at physiological maturity and geminate immediately. They also do not survive in low moisture environments and display issues with seed viability (Farnsworth, 2000; Benech-Arnold, 2004; Ceccato et al., 2015; McGinty et al., 2021).

In the current study, ABA dose-response curves were used to make initial dormancy groupings, and then hormone screening with ABA and GA was used to establish a baseline for dormancy type and strength under controlled conditions. In the evaluation of 189 quinoa varieties, the majority of varieties tested, 140 in total, displayed no observable dormancy (ND) (**Figure 1**; **Supplemental Table 1**). All ND varieties reached nearly complete germination regardless of imbibition treatment. Although ND varieties displayed at least one hallmark of recalcitrance, absence of primary dormancy at physiological maturity, it is interesting to note that dry seed moisture for all varieties was less than 10%. If the ND seeds were truly recalcitrant the expectation would be that they would not survive storage in the low moisture conditions of the current study and would fail to germinate. Future work will need to better characterize embryo maturation in the ND varieties to determine if any display a period of embryo quiescence after maturation and prior to germination, the hallmarks of seed dormancy. It will also be important to evaluate long-term viability for ND seeds to better understand seed longevity in the group.

In addition to identifying ND varieties, it was determined that primary (physiological) dormancy ranging from strong (SD) to weak (WD) was present in the remaining 48 of the total varieties tested (**Figure 1**; **Supplemental Table 1**). SD varieties had germination percentages less than 25% in the absence of ABA, and even lower, and in many instances zero, in presence of 10 µM ABA. Additionally, SD varieties displayed a high degree of GA-insensitivity, with low germination rates regardless of treatment (**Figure 2**). The current study also determined that MD varieties displayed a significantly greater response to GA compared to NT and ABA treatments at physiological maturity reaching > 50% germination (**Figure 3**). Without hormone, germination percentages for MD varieties remained < 50% and these varieties retained ABA-sensitivity. Finally, varieties with weak dormancy (WD) at physiological maturity reached > 75% germination with GA, > 50% germination with NT, and displayed increased ABA-insensitivity (**Figure 4**).

As mentioned above, the absence of germination may be an indication of strong primary dormancy or issues with seed viability. To confirm that dormancy was the cause of reduced rates of germination in all SD and some MD lines additional dormancy breaking treatments, after-ripening and scarification, were tested. One month of after-ripening rescued seed germination in 7 of 12 SD and all MD varieties which reached > 75% germination when imbibed without hormone or in the presence of 10 µM GA (**Figures 5A, B**). Of the remaining one-month after-ripened SD varieties that did not reach > 75% germination, two retained a similar level of ABA-sensitivity and germination percentages without hormone but showed very different germination profiles on GA (**Figure 5A**), indicating differences in rates of dormancy loss (**Figure 5A**). Additionally, it was determined that scarification was needed to rescue germination in two poorly germinating varieties, and a period of after-ripening longer than a month was required to rescue germination in three remaining non-germinating SD lines (**Figures 6A, B**). Collectively, these results are interesting because they indicate the existence of dormant quinoa varieties, and variation in the time to after-ripen. They also suggest that in addition to embryo-imposed seed dormancy consistent with what was reported by Ceccato et al. (2015), certain quinoa varieties may also have seed coat-imposed dormancy. Interestingly, Ceccato et al. (2015) also found that in the field, sowing date influenced transitions between seed coat and embryo-imposed seed dormancy. Future work will need to further characterize embryo and seed coat-imposed dormancy in quinoa populations and the impact of environment on dormancy (Penfield and MacGregor, 2017). Varieties that display consistent dormancy phenotypes, maybe be useful varieties to breed for improved PHS tolerance. Further, research will also need to develop a quinoa panicle wetting tests to evaluate if dormant varieties identified, are also resistant to PHS, and the impact of panicle morphology on PHS tolerance or susceptibility.

### 4.2 Morphological and agronomic variables with impacts on seed dormancy

Based on previous research by Ceccato et al. (2015), it was initially hypothesized that darker and thicker seed coats would be associated with more seed dormancy. However, a limitation of the Ceccato study was that only two varieties were tested. In the current study, seed coat thickness and color were evaluated in a much larger panel of quinoa; in total 181 of 189 varieties used for dormancy screens. Interestingly, of the dark-coated seeds, i.e., black or brown, only three of 37 varieties fell into the SD and MD categories, and the percentage of black or brown seed coats within a given dormancy category, was equal or more abundant in WD and ND varieties (**Figure 7**). However, it was determined using FAMD and OLR analyses that our hypothesis was partially supported. While the FAMD analysis showed weak clustering indicative of a possible association between seed coat color, seed coat thickness, and dormancy strength, as well as between dormancy and seed moisture and protein (**Figures 8B**, **9B**), OLR analysis showed that only seed coat thickness had a categorical effect on dormancy strength (**Table 2**). Given that both dark and light-colored seed coats were found for quinoa varieties in all dormancy categories (SD-WD) as well as in ND varieties, this result was not surprising and demonstrated that across a large set of quinoa varieties, darker seed coat color did not appear to be as predictive of quinoa seed dormancy as it is in other seeds, including cereals (Penfield and Macgregor, 2017; Vetch et al., 2019). On the other hand, although average seed coat thickness was similar across all dormancy categories, the varieties with the thickest seed coats were among the most dormant at physiological maturity. For example, variety D-11924 had the thickest seed coat of all varieties tested and is consistent with the previous findings by Ceccato et al. (2015) (**Table 1**; **Supplemental Table 2**). Therefore, selecting quinoa varieties with thicker seeds coats may also result in greater seed dormancy and by extension perhaps PHS-tolerance. Future work will need to evaluate the relationship between seed coat thickness and PHS-tolerance.

Other morphological and agronomic variables were also included in this study to evaluate a possible role in quinoa seed dormancy. Specifically, we focused on a set of secondary seed morphological characteristics linked to seed shape, including area, perimeter, major and minor axis length, and eccentricity (**Figure 8**; **Tables 2**-**4**). While significant correlations were not detected between these variables using FAMD analysis, OLR analysis of the same variables indicated that eccentricity had an impact on dormancy strength (**Table 4**). Eccentricity describes the curvature of the seed, and like seed coat thickness may be impacted by environmental growing conditions (Balkaya and Odabas, 2002; Cervantes et al., 2016). Unlike seed coat thickness, eccentricity was determined using a non-destructive higher through-put imaging technique; a powerful tool for selection when sample size is limited. Eccentricity scores range between zero and one and seeds with eccentricity scores closer to zero are more circular, and seeds with eccentricity scores closer to one are more elongated (Balkaya and Odabas, 2002; Cervantes et al., 2016). Although eccentricity may not be a universal feature impacting dormancy in all seed types, previous studies in maize and weeds, including *Chenopodium album*, demonstrate that elongated seeds, or seeds with higher eccentricity scores were less dormant, whereas those that were rounder and had lower eccentricity scores were more dormant (Adebisi et al., 2005; Gardarin and Colbach, 2015). As with seed coat thickness, in the current study, average eccentricity values were remarkably similar across dormancy categories. However, eccentricity scores closer to one were associated with ND-varieties, and SD-varieties had a narrower range of eccentricity scores closer to zero (**Table 1**; **Supplemental Table 2**). Among the SD-varieties only two had eccentricity scores above 0.4; Ames-13721 and CHEN-291 with scores of 0.435 and 0.499, respectively. In contrast, of the seven varieties with eccentricity scores above 0.6, five were ND- and two were WD-varieties. This result suggests that another way to improve selection for greater primary dormancy in quinoa may be to avoid samples with eccentricity values greater than 0.5. Future work will need to investigate the limits of using eccentricity as a possible metric for evaluating seed dormancy in quinoa especially in the context of growing environment.

## 5 Conclusions

### 5.1 Implications for breeding for increased seed dormancy in quinoa

In the current study, dormancy screening methods, FAMD models and OLR analyses were developed to establish a baseline for quinoa primary seed dormancy under controlled conditions, and then to identify possible morphological and agronomic variables that may influence seed dormancy in a subset of the quinoa world core collection (Baskin and Baskin, 2004; Finch-Savage and Leubner-Metzger, 2006; Tuttle et al., 2015; McGinty et al., 2021). Hormone screening is a useful and efficient way to identify the presence or absence of primary dormancy, and the strength or primary dormancy at physiological maturity. Hormone screening used in this study identified forty-eight quinoa varieties with some level of primary dormancy at physiological maturity and one hundred forty-one with no apparent primary dormancy at physiological maturity. Identification of the presence of primary dormancy is important because seeds that have and retain primary dormancy may be coaxed back into a state of secondary dormancy, whereas seeds that lack dormancy to begin with or that have completely lost dormancy cannot (Bewley, 1997). Of the forty-eight varieties with primary dormancy, twelve had strong primary dormancy resulting from two different mechanisms; embryo- and seedcoat-imposed dormancy consistent with findings in Ceccato et al. (2015). While the results from this study indicate that strong primary dormancy is uncommon, representing less than 10% of the varieties tested, the SD varieties identified hold the greatest potential for breeding for increased seed dormancy and future quinoa varieties that are less prone to PHS. It is also encouraging that at least 25% of the total varieties tested showed some level of primary dormancy at maturity suggesting that under the right conditions it may be possible to optimize dormancy strength.

In addition to hormone screens a phenetic modeling approach was used to identify other agronomically important variables that might contribute to quinoa seed dormancy. Categories derived from dormancy screening, i.e., SD, MD, WD, and ND were used in both FAMD and OLR models. FAMD modeling was used first to identify associations between the variables evaluated. OLR models (Agresti, 2002) were then used to highlight combined variable relationships and to tease apart the effect of singular variables that may contribute to dormancy.

Based on FAMD and OLS modeling it was determined that seed coat thickness and eccentricity may be important variables that impact quinoa seed dormancy. Additionally, it was determined that while there is a strong relationship between seed coat color and dormancy strength in other crops like wheat, seed coat thickness is a better predictor of primary dormancy in quinoa.

## Data availability statement

The original contributions presented in the study are included in the article/**Supplementary Material**, further inquiries can be directed to the corresponding authors.

## Author contributions

EM and AH designed experiments; EM, EC, NM, and AH were responsible for data collection and analysis. EM, EC, NM, CO-G, ES, KM and AH drafted the manuscript. All authors contributed to the article and approved the submitted version.

## Supplementary material

Supplementary Table 1 ABA-dose responses and assigned dormancy categories for 189 quinoa varieties at physiological maturity.

Supplementary Table 2 Seed coat thickness, color, and composition measurements for 181 quinoa varieties.
